# NovoSorb Biodegradable Temporizing Matrix (PolyNovo) for Aesthetic Nasal Reconstruction: A Case Report of Rhinophyma Resurfacing

**DOI:** 10.7759/cureus.87678

**Published:** 2025-07-10

**Authors:** Ioannis Kyriazidis, Efterpi Demiri, Alexandra Lazaridou, Leonidas Pavlidis, Athanasios Papas

**Affiliations:** 1 Department of Plastic and Reconstructive Surgery, General Hospital Papageorgiou, Thessaloniki, GRC; 2 Department of Plastic and Reconstructive Surgery, School of Medicine, Faculty of Health Sciences, Aristotle University of Thessaloniki, Thessaloniki, GRC

**Keywords:** aesthetic surgery, biodegradable scaffold, case report, dermatology case report dlqi (dermatology life quality index), dlqi, face-q, face-q scale, nasal reconstruction, novosorb, polynovo

## Abstract

Rhinophyma, a severe form of rosacea, causes significant nasal disfigurement, posing challenges for aesthetic reconstruction after excision. This case report details the novel application of PolyNovo’s NovoSorb^®^ biodegradable temporizing matrix (BTM), a synthetic dermal substitute, for aesthetic nasal reconstruction following rhinophyma excision and utilizing secondary intention healing. A 65-year-old male with progressive rhinophyma underwent tangential excision and burring. A tailored piece of NovoSorb^®^ BTM was applied and secured to the defect. Postoperative care involved paraffin gauze. The patient exhibited excellent aesthetic results with minimal scarring. Outcome measures, including the FACE-Q Skin Cancer Module and the Dermatology Life Quality Index (DLQI), demonstrated significant improvements in appearance-related concerns, psychosocial well-being, and daily functioning. This report highlights the innovative use of NovoSorb^®^ BTM and secondary intention healing for rhinophyma reconstruction. The successful outcome, supported by validated patient-reported outcome measures, suggests that BTM may be a valuable tool in similar cases, offering a new avenue for achieving both functional and aesthetic goals.

## Introduction

Rhinophyma is a disfiguring condition characterized by sebaceous gland hyperplasia and fibrosis, primarily affecting the nasal tip and alae [1]. While surgical excision is often necessary, achieving aesthetically pleasing reconstruction remains challenging [2]. Traditional reconstructive approaches have relied on various techniques, including secondary intention healing, skin grafting, and local flaps, each with inherent limitations, such as prolonged healing times, donor site morbidity, and unpredictable aesthetic outcomes.

PolyNovo NovoSorb^®^ biodegradable temporizing matrix (BTM) (PolyNovo Biomaterials Pty Ltd, Melbourne, Australia), a bilaminar biodegradable synthetic dermal matrix, has gained increasing clinical acceptance for managing complex wounds, having been originally developed for burn injury treatment [3]. Clinical applications of BTM have subsequently broadened beyond its initial indication to encompass reconstruction of upper and lower extremity defects [4-6], scalp reconstruction [7-9], and various other wound pathologies [10]. The biodegradable polyurethane foam component undergoes hydrolytic degradation while functioning as a structural scaffold that facilitates vascular tissue integration, ultimately forming a neodermis. The non-biodegradable polyurethane seal layer, which adheres to the superior surface to provide wound temporization and prevent desiccation, is removed following successful foam integration. Typically, once adequate vascular tissue ingrowth into the foam matrix has occurred, a split-thickness skin graft is harvested and applied to the prepared wound bed.

Nevertheless, emerging clinical evidence has demonstrated the feasibility of utilizing BTM as a definitive dermal substitute without subsequent skin grafting, permitting wound closure through secondary intention healing. Several individual case reports have documented successful healing by secondary intention over BTM [9,11-13]. These reported cases involved wounds that achieved complete epithelialization directly over the BTM substrate. Although prior case series have reported BTM utilization with secondary intention healing for managing small, complex nasal defects following elective skin malignancy excision [13], the application of this technique to rhinophyma resurfacing constitutes a novel therapeutic strategy. While the literature describes a very limited number of cases of rhinophyma reconstruction using dermal substitutes [14-16], significant gaps remain regarding the specific application of NovoSorb^®^ BTM for rhinophyma resurfacing. The unique characteristics of rhinophymatous tissue, including its highly vascularized nature, irregular surface topography, and potential for recurrence, present distinct challenges that may differ from post-oncological reconstruction scenarios. Furthermore, the aesthetic expectations and functional requirements for rhinophyma patients may vary considerably from those undergoing cancer reconstruction, necessitating tailored outcome measures and treatment protocols.

This report presents a novel application of NovoSorb^®^ BTM, a synthetic dermal substitute, for aesthetic nasal resurfacing following rhinophyma excision utilizing secondary intention healing. To our knowledge, this is the first case report of a single-stage, graft-free approach to rhinophyma resurfacing using NovoSorb^®^ BTM, offering a potentially less invasive option that eliminates donor site morbidity while achieving excellent aesthetic outcomes.

The patient gave informed consent for publication of this case report and accompanying images. The patient provided explicit consent for publication of his medical information in anonymized form, use of clinical photographs showing his condition before and after treatment, sharing of his treatment experience and outcomes for educational purposes, and publication in medical literature and potential use in medical education. Consent documentation is available upon request from the corresponding author.

## Case presentation

Patient information

A 65-year-old male, a non-smoker, presented with a five-year history of progressive nasal disfigurement diagnosed as rhinophyma. He had been receiving treatment for hypertension, without antiplatelet agents. The patient's main concerns included significant nasal disfigurement affecting his appearance and self-confidence, social embarrassment leading to withdrawal from social activities, and psychological distress related to his facial appearance. Medical, family, and psychosocial history, including relevant genetic information, was as follows: the patient had a medical history of hypertension, well-controlled with antihypertensive medications; there was no family history of rosacea or similar skin conditions.

The patient reported a significant impact on his quality of life, with avoidance of social situations and decreased participation in professional activities due to self-consciousness about his appearance. The patient had not undergone any previous surgical interventions for his rhinophyma. Conservative management with topical treatments had been unsuccessful in controlling the progression of the condition.

Clinical findings

**Figure 1:**
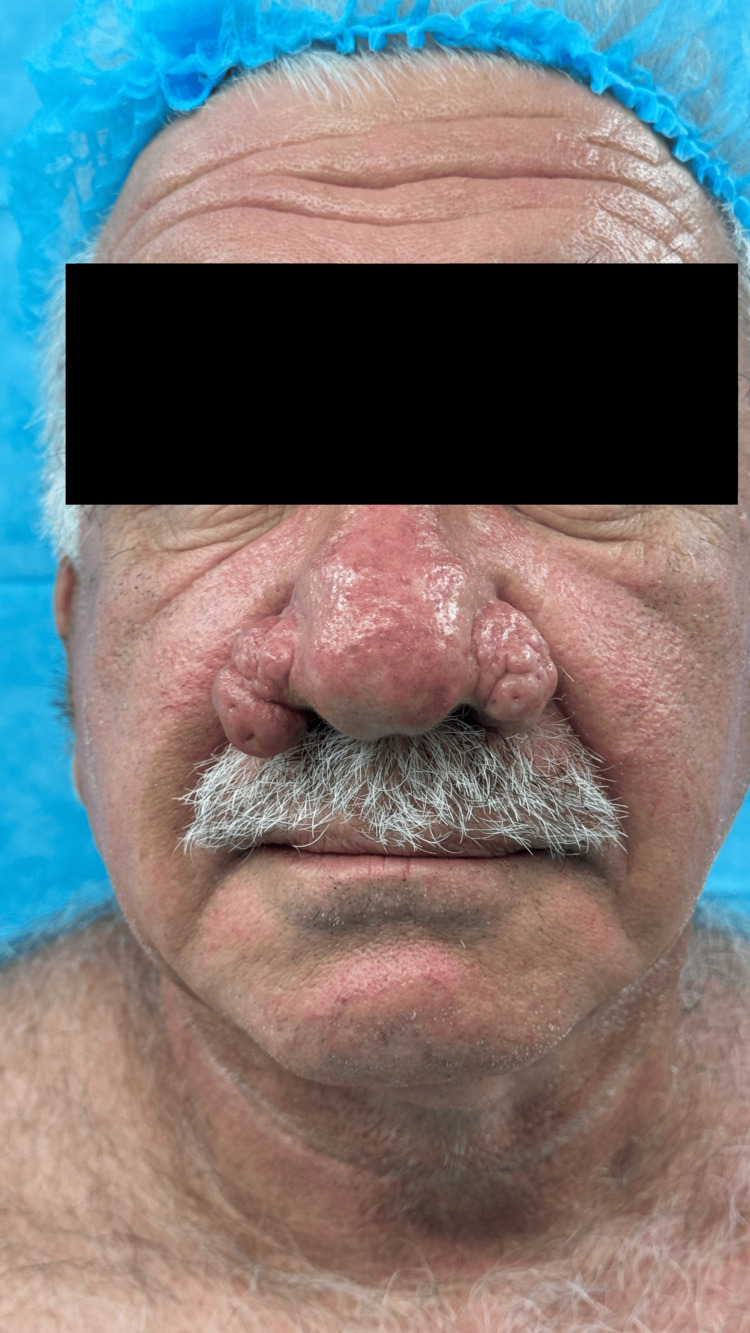
Preoperative en-face view demonstrating significant nasal disfigurement due to advanced rhinophyma

**Figure 2:**
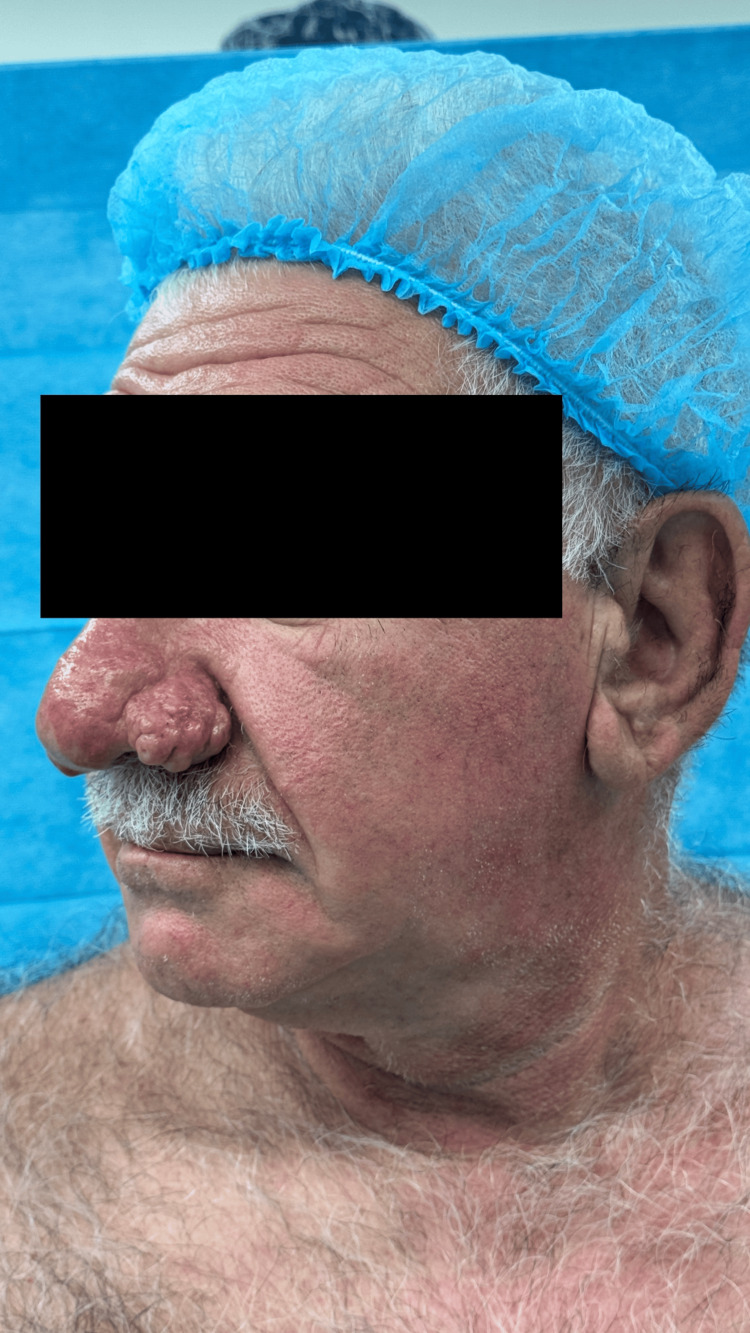
Preoperative left lateral view highlighting the extent of rhinophyma affecting the nasal profile

Significant physical examination findings demonstrated characteristic features of advanced rhinophyma with significant hypertrophy and nodular changes affecting the nasal tip and alae. The nasal skin showed marked thickening with a lobulated, irregular surface texture. The affected areas demonstrated the typical "cobblestone" appearance with enlarged pores and sebaceous gland hyperplasia. The nasal contour was severely distorted, with loss of normal anatomical landmarks. Nasal airway patency was not compromised, and internal nasal examination revealed no significant intranasal pathology (Figures 1, 2). Sensation over the affected areas was intact.

Diagnostic assessment

The diagnosis was established through comprehensive clinical evaluation, including detailed medical history, physical examination, clinical photography for documentation and treatment planning, assessment of functional impact on nasal breathing, and evaluation of psychosocial impact using validated questionnaires. Several diagnostic considerations were encountered during the evaluation process. Determining the optimal extent of tissue excision while preserving functional nasal anatomy required careful preoperative planning. The irregular surface topography made it challenging to predict the depth of excision needed for complete removal of the affected tissue. Distinguishing between external nasal obstruction due to rhinophyma versus intranasal pathology required thorough examination to ensure appropriate treatment planning. Quantifying the psychosocial impact of the condition necessitated the use of validated instruments to establish baseline measurements for outcome assessment.

The differential diagnosis included several conditions. Basal cell carcinoma was considered given the nodular appearance and chronic nature, particularly in areas of ulceration or rapid growth; however, the bilateral symmetric involvement and characteristic appearance supported the diagnosis of rhinophyma. Sebaceous hyperplasia was considered, but the extent and severity of tissue involvement clearly indicated advanced rhinophyma rather than simple sebaceous hyperplasia. Chronic actinic damage and solar elastosis can cause nasal skin thickening, but the characteristic lobulated appearance and sebaceous gland involvement supported rhinophyma diagnosis. Granulomatous conditions such as sarcoidosis or granulomatous rosacea were considered but ruled out based on the clinical presentation and absence of systemic involvement. Ultimately, a diagnosis of advanced rhinophyma with significant functional and psychosocial impact was established. The patient's age, non-smoking status, absence of comorbidities affecting wound healing, and good baseline health suggested a favorable prognosis for surgical intervention and healing.

Therapeutic intervention

The patient underwent the procedure under local anesthesia. Tangential excision and burring of the rhinophymatous tissue were performed. A tailored piece of NovoSorb^®^ BTM was applied to the defect and secured with through-and-through sutures incorporating paraffin-soaked gauze as a bolster. The surgical technique involved local anesthesia with lidocaine and epinephrine, tangential excision of rhinophymatous tissue using electrocautery and surgical burring to achieve smooth contours, and precise fitting of NovoSorb^®^ BTM to the defect.

**Figure 3:**
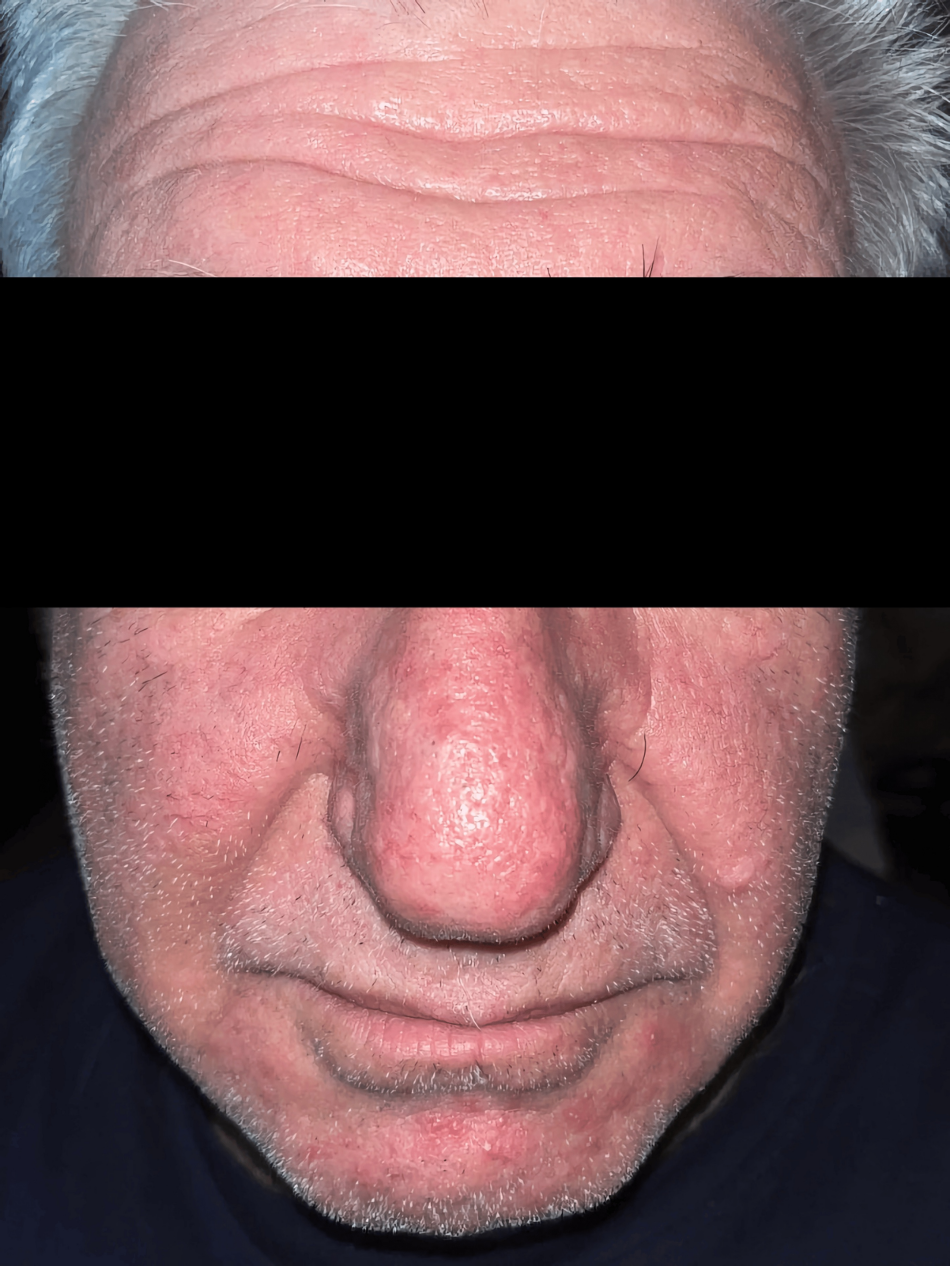
Postoperative en-face view at six months demonstrating a natural-looking nasal contour, excellent color match, and minimal scarring following rhinophyma excision and reconstruction with NovoSorb® BTM and secondary intention healing BTM: biodegradable temporizing matrix

**Figure 4:**
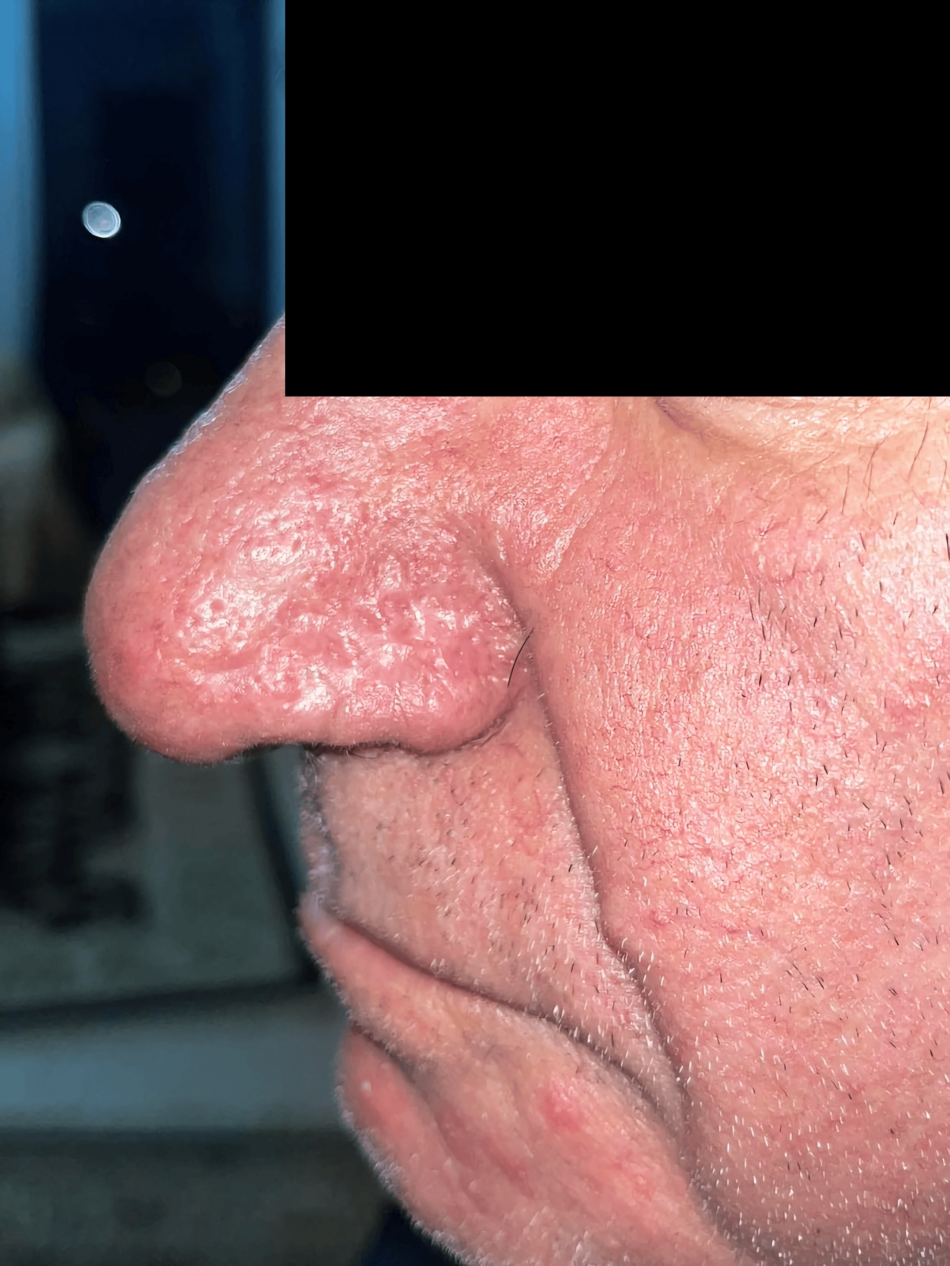
Postoperative left lateral view at six months showing restoration of a natural nasal profile with excellent aesthetic outcome

Postoperatively, the patient reported minimal discomfort. The paraffin gauze bolster was removed at five days postoperatively. The area was cleaned with chlorhexidine only if fluid collection was observed beneath the outer silicone sheet. Weekly follow-up demonstrated progressive healing without complications. This approach is more costly than traditional approaches but offers significant advantages in terms of eliminating donor site morbidity and achieving excellent aesthetic outcomes (Figures 3, 4).

Follow-up and outcomes

Weekly follow-up demonstrated excellent integration and delamination of BTM at five weeks, with complete epithelialization achieved 10 weeks postoperatively. At six months postoperatively, the reconstructed area exhibited a natural-looking nasal contour, soft skin on palpation, and excellent color match, demonstrating a highly satisfactory aesthetic outcome.

Patient-reported outcomes were assessed using the FACE-Q Skin Cancer Module [17] and the Dermatology Life Quality Index (DLQI) [18]. On the FACE-Q Appearance-Related Distress scale, the patient scored 42 preoperatively and 18 postoperatively, demonstrating a substantial improvement. Preoperatively, the patient scored 38 on the Social Function scale, which improved to 55 postoperatively. Regarding psychological well-being, the patient's score improved from 35 preoperatively to 60 postoperatively. The Satisfaction with Outcome scale score increased from 25 preoperatively to 85 postoperatively, reflecting a high level of satisfaction. The overall DLQI score decreased from 15 preoperatively to 3 postoperatively, indicating a considerable reduction in the impact of the condition on the patient's quality of life.

The patient demonstrated excellent adherence to postoperative care instructions. Tolerability was excellent with minimal discomfort reported throughout the healing process. No issues with wound care compliance or follow-up attendance were encountered. No adverse events were encountered during the treatment course. No complications related to BTM application or integration occurred. No signs of infection, delayed healing, or material rejection were observed. Healing progressed as anticipated without any unexpected delays. Sensation remained intact over the reconstructed area. The skin quality demonstrated a soft, pliable texture with an excellent color match to the surrounding tissue.

Timeline

A timeline of the patient's disease and treatment course is presented in Table 1.

**Table 1:** A timeline of the patient's disease and treatment course BTM: biodegradable temporizing matrix

Time point	Event	Clinical details
5 years prior to presentation	Initial symptom onset	Patient first noticed nasal skin changes and progressive thickening
2 years prior to presentation	Formal diagnosis	Diagnosis of rhinophyma established by dermatologist
6 months prior to presentation	Conservative treatment failure	Topical treatments unsuccessful; surgical consultation sought
Day 0 (preoperative)	Baseline assessment	Clinical photographs obtained, patient-reported outcome measures administered
Day 0 (operative)	Surgical intervention	Tangential excision and burring under local anesthesia; NovoSorb^®^ BTM application with paraffin gauze bolster
Postoperative day 1	Immediate postoperative period	Patient reported minimal discomfort; dressing intact
Postoperative day 5	First follow-up visit	Paraffin gauze bolster removed; excellent BTM integration observed
Postoperative week 2	Early healing phase	Progressive epithelialization from wound edges; no complications noted
Postoperative week 5	BTM integration phase	Excellent integration and delamination of BTM demonstrated
Postoperative week 10	Complete healing achieved	Complete epithelialization accomplished
Postoperative month 6	Final assessment	Excellent aesthetic outcome documented; patient-reported outcome measures reassessed

## Discussion

While BTM is well-established in burns and limb reconstruction [19,20], its use in aesthetic nasal resurfacing is novel. The complex anatomical structure of the nose, composed of cartilage and limited surrounding skin, presents a significant reconstructive challenge. Traditional approaches, such as full-thickness skin grafts, can lead to well-known issues like contour irregularities, color-texture mismatch, and donor site morbidity. Local flaps, while offering potential advantages, require more technical expertise and may necessitate multiple stages. The use of BTM, particularly with secondary intention healing, offers a compelling alternative that addresses these limitations. BTM's porous structure allows for rapid vascularization and tissue integration, creating a neodermis that supports epithelialization [21]. This eliminates the need for a separate donor site, as required with skin grafts, thus avoiding potential complications and morbidity at that site. Furthermore, the gradual biodegradation of BTM and its replacement by the patient's own tissue contribute to a natural-looking result, addressing the common concerns of color and texture mismatch often associated with skin grafts. This approach also minimizes the patient's anesthetic and surgical time, which can be particularly beneficial for older patients or those with comorbidities.

While the literature describes a very limited number of cases of rhinophyma reconstruction using dermal substitutes [14-16], to our knowledge, this is the first documented case utilizing NovoSorb^®^ BTM (PolyNovo) for this specific purpose. The use of acellular biologic tissue matrices for rhinophyma reconstruction has been described, with one of the shortcomings of that treatment being the unpredictable thickness of the resulting neodermis. Moreover, unlike these previous reports [14-16] that lacked objective patient-reported outcome measures and sometimes noted aesthetic concerns [15], our case demonstrates successful rhinophyma resurfacing with BTM and secondary intention healing, supported by quantifiable improvements in validated FACE-Q and DLQI scores.

Our case builds upon the emerging evidence for NovoSorb^®^'s utility in nasal reconstruction, notably demonstrated very recently by McMahon et al. [13] in their series of 32 patients with post-oncological defects. While their work focused on smaller defects of the nasal dorsum and sidewall following skin cancer excision, our case extends this application to the more challenging context of rhinophyma, often involving extensive tissue involvement across multiple nasal subunits. Despite differences in etiology, defect complexity, and outcome measures, both studies confirm the effectiveness of secondary intention healing over BTM for achieving significant aesthetic improvements and patient satisfaction without skin grafting. To our knowledge, McMahon et al.'s paper is the only one that currently discusses the use of NovoSorb^®^ with secondary intention healing, for nasal reconstruction, and we would like to expand on this. The consistent findings across different patient populations and defect types, combined with the specific advantages of BTM demonstrated in our rhinophyma case (eliminating donor site harvesting, enabling precise contouring, and high patient satisfaction), support its consideration as a valuable option in a broader range of nasal reconstructive scenarios.

The successful outcome in this case, supported by quantifiable improvements in validated outcome measures, suggests that NovoSorb^®^ BTM with secondary intention healing represents a viable option for rhinophyma resurfacing. This report demonstrates the successful application of NovoSorb^®^ BTM for aesthetic nasal resurfacing following rhinophyma excision. The results, supported by FACE-Q and DLQI assessments, suggest that BTM may be a valuable tool in achieving both functional and aesthetic goals in similar cases. Further research, including larger case series, is needed to validate these findings and establish long-term efficacy. To our knowledge, this is the first case report of a single-stage, graft-free approach to rhinophyma resurfacing using Novosorb BTM, highlighting a potentially less invasive option that prioritizes patient comfort, minimizes donor site morbidity, and delivers excellent aesthetic results.

Limitations of this report include its nature as a single case study and the relatively short follow-up period (six months). However, the compelling outcome, supported by validated patient-reported outcome measures (FACE-Q and DLQI), adds to the limited body of literature on the topic and highlights the need for further investigations.

## Conclusions

This report demonstrates the successful application of NovoSorb^®^ BTM for aesthetic nasal resurfacing following rhinophyma excision. The results, supported by FACE-Q and DLQI assessments, suggest that BTM may be a valuable tool in achieving both functional and aesthetic goals in similar cases. Further research, including larger case series, is needed to validate these findings and establish long-term efficacy. To our knowledge, this is the first case report of a single-stage, graft-free approach to rhinophyma resurfacing using NovoSorb^®^ BTM, highlighting a potentially less invasive option that prioritizes patient comfort, minimizes donor site morbidity, and delivers excellent aesthetic results.
